# Complete chloroplast genome sequences of *Dioscorea*: Characterization, genomic resources, and phylogenetic analyses

**DOI:** 10.7717/peerj.6032

**Published:** 2018-12-04

**Authors:** Zhenyu Zhao, Xin Wang, Yi Yu, Subo Yuan, Dan Jiang, Yujun Zhang, Teng Zhang, Wenhao Zhong, Qingjun Yuan, Luqi Huang

**Affiliations:** 1 State Key Laboratory Breeding Base of Dao-di Herbs, National Resource Center for Chinese Materia Medica, China Academy of Chinese Medical Sciences, Beijing, China; 2 Tianjin University of Traditional Chinese Medicine,Tianjin, China; 3 Infinitus (China) Company Ltd, Guangzhou, China; 4 Department of Immunology, Medical College, Wuhan University of Science and Technology, Wuhan, China; 5 School of Chinese Materia Medica, Beijing University of Chinese Medicine, Beijing, China; 6 National Cancer Center/National Clinical Research Center for Cancer/Cancer Hospital, Chinese Academy of Medical Sciences and Peking Union Medical College, Beijing, China

**Keywords:** Chloroplast genome, Dioscorea, Phylogeny, Single sequence repeats, Variable marker

## Abstract

*Dioscorea* L., the largest genus of the family *Dioscoreaceae* with over 600 species, is not only an important food but also a medicinal plant. The identification and classification of *Dioscorea* L. is a rather difficult task. In this study, we sequenced five *Dioscorea* chloroplast genomes, and analyzed with four other chloroplast genomes of *Dioscorea* species from GenBank. The *Dioscorea* chloroplast genomes displayed the typical quadripartite structure of angiosperms, which consisted of a pair of inverted repeats separated by a large single-copy region, and a small single-copy region. The location and distribution of repeat sequences and microsatellites were determined, and the rapidly evolving chloroplast genome regions (*trnK-trnQ*, *trnS-trnG*, *trnC-petN*, *trnE-trnT*, *petG-trnW-trnP*, *ndhF*, *trnL-rpl32*, and *ycf1*) were detected. Phylogenetic relationships of *Dioscorea* inferred from chloroplast genomes obtained high support even in shortest internodes. Thus, chloroplast genome sequences provide potential molecular markers and genomic resources for phylogeny and species identification.

## Introdution

*Dioscorea* L. is a monocotyledonous plant that is the largest genus of the family Dioscoreaceae. It comprises more than 600 plant species, almost all of which are distributed in Southeast Asia, Africa, Central America, South America, and other tropical or subtropical regions of the world, while a few occur in Europe and North America (Caddick et al., 2002; Hsu et al., 2013). Some *Dioscorea* species are an economically important supply of starch in the staple diet such as *D. alata*, *D. esculenta*, *D. cayenensis*, *D. dumetorum*, and *D. rotundata*. The genus is also a favored source of medicinal plants, such as *D. nipponica, D. opposita*, and *D. zingiberensis*. (Zhai et al., 2009).

The identification of *Dioscorea* L. has presented a challenge to systematists because of its great morphological variations, especially the aerial parts, such as leaves (Wilkin et al., 2005). Furthermore, its hypanthium is relatively small and dioecious, which makes the classification of *Dioscorea* L. into a rather difficult task (Hsu et al., 2013; Wilkin et al., 2005). For phylogenetic studies of *Dioscorea*, some chloroplast molecular markers (such as *rbcL*, *matK*, *trnH-psbA*, *trnL-F*), have been analyzed (Gao et al., 2008; Hsu et al., 2013; Wilkin et al., 2005). Although molecular markers provide some information for the taxonomy of *Dioscorea*, phylogenetic analyses are low resolution due to these limited data. Further studies to seek high resolution molecular markers in the species level to the success of identification and phylogeny in *Dioscorea* is necessary. Four complete *Dioscorea* chloroplast genomes (*D. elephantipes*, *D. rotundata*, *D. villosa*, and *D. zingiberensis*) have been released in GenBank (Hansen et al., 2007; Mariac et al., 2014), which provides opportunities to develop more genetic resources for discriminating between species and populations. The chloroplast genome in angiosperms has a typical quadripartite structure, with two copies of inverted repeats (IRs) separating the large single-copy (LSC) and small single-copy regions (SSC), and the genome size ranging from 120 to 170 kb in length. Because of maternal inheritance, low rates of nucleotide substitutions and very low recombination, chloroplast DNA sequences have often been used for phylogenetic studies of higher plants in order to resolve complex evolutionary relationships (Burke et al., 2016; Dong et al., 2017). Comparisons of chloroplast genomes provide additional effective resources for the development of variable markers, which are used for phylogeny or species identification (Dong et al., 2012). Next Generation Sequencing (NGS) technique generates the large numbers of DNA sequences at relatively low cost and promptly extended gene-based phylogenetics to phylogenomics.

Here, we investigated the complete chloroplast genomes of five *Dioscorea* species through NGS and compared them with four previously sequenced species. The comparative analysis of nine complete *Dioscorea* chloroplast genomes was conducted to demonstrate the features and structural differentiation of the sequences, also to provide valuable chloroplast molecular markers for further phylogenetic and species identification. Furthermore, we tested the feasibility of phylogeny reconstruction using chloroplast genome data.

## Materials and Methods

### Sample materials and DNA extraction

Young leaves of five *Dioscorea* species were harvested from Lijiang, Yunan, China (*D. aspersa*), Lushan, Sichuan, China (*D. alata*), Lin’an, Zhejiang, China (*D. bulbifera*), and Minhou, Fujian, China (*D. futschauensis* and *D. polystachya*). Voucher specimens were deposited in the herbaria of CMMI (Institute of Chinese Materia Medica, China Academy of Chinese Medical Sciences). Total genomic DNA was extracted from silica-dried leaves following the method of Li et al. protocol (2013). Then, DNA was purified by the Wizard DNA CleanUp System (Promega, Madison, WI, USA). Final DNA quality was assessed on spectrophotometry, and their integrity was evaluated using a 1% (w/v) agarose gel.

### Illumina sequencing, assembly, and annotation

DNA was sheared to fragments of 400–600 bp with an ultrasonic disruptor. An Illumina paired-end library was constructed with the NEBNext® Ultra^™^ DNA Library Prep Kit according to the manufacturer’s protocol. Paired-end sequencing was conducted on an Illumina HiSeq X 10 platform. For each species, approximately 10.0 Gb of raw data were generated with pair-end 150 bp read length. A four-step approach was employed to assemble the chloroplast genome. First, raw sequence reads were filtered for primer/adaptor sequences and low-quality reads with the NGS QC Tool Kit (Patel & Jain, 2012). By using the assembly program SPAdes 3.6.1 (Bankevich et al., 2012), with parameters, kmer = 95, contigs were generated from high quality paired-end reads. Second, chloroplast genome sequence contigs were selected from the initial assembly by performing a BLAST search using the *D. elephantipes* chloroplast genome sequence as a reference (GenBank accession number: EF380353). A high copy number of extranuclear DNA was present in the total DNA, usually around 5–10% of chloroplast DNA and around 1–2% of mitochondrial DNA. After the de novo assembling, the coverages of the contigs are significantly different among three genomes. Coverage of chloroplast contigs are much higher than those in nuclear and mitochondrial genome. In this study, we also used this method to select chloroplast genome contigs. The selected contigs from chloroplast genomes were further assembled using Sequencher 5.4.5. Third, ambiguous nucleotides or gaps and the four junctional regions between the IRs and SSC/LSC in the chloroplast genome sequences were further confirmed by PCR amplification and Sanger sequencing with specific primers (Dong et al., 2013). Finally, clean reads were remapped to the draft genome sequences and yielded the sequences.

Three methods were used to check the assembling accuracy of chloroplast genome sequence. First, half of the amount of raw data was used to assemble the chloroplast genome; Second, original reads were assessed through strict quality control and then only high quality reads were filtered to assemble contigs. The third method was a four-step approach in this method. Three methods gave completely identical result of assemblage chloroplast genome sequence. Furthermore, the Geneious 11.1.2 was used to map all reads to the assembled chloroplast genome sequence. The consensus sequences were produced using mapped reads in Geneious.

Dual Organellar GenoMe Annotator (DOGMA) using the default parameters was used to annotate chloroplast genome sequences (Wyman, Jansen & Boore, 2004). BLASTX and BLASTN searches were utilized to accurately annotate the genes encoding proteins and the locations of the transfer RNAs (tRNAs). The Genome Vx software was used to draw a chloroplast genome map (Conant & Wolfe, 2008).

### Analysis of tandem repeats and single sequence repeats

Three types of repeat sequences were identified in the *Dioscorea* chloroplast genome. We used REPuter to identify dispersed and palindromic repeats (Kurtz et al., 2001). The minimum similarity percentage of two repeat copies was limited to 90%, the minimum repeat size was 30 bp, and the hamming distance was 3. Tandem repeats were detected by Tandem Repeats Finder (https://tandem.bu.edu/trf/trf.html), with two, five, and seven set for the alignment parameters match, mismatch, and indel, respectively.

Single sequence repeats (SSRs) were detected by MISA (MIcroSAtellite; http://pgrc.ipk-gatersleben.de/misa) with the search parameters set at >10 repeat units for mononucleotide, >5 repeat units for dinucleotide, >4 repeat units for trinucleotide, and >3 repeat units for tetranucleotide, pentanucleotide, and hexanucleotide SSRs.

### Comparison whole chloroplast genomes and divergent hotspot identification

The mVISTA program (http://genome.lbl.gov/vista/mvista/submit.shtml) with Shuffle-LAGAN mode (Frazer et al., 2004) was used to compare the *Dioscorea* chloroplast genomes. *D. elephantipe*s chloroplast genome was used as reference. All *Dioscorea* sequenced chloroplast genomes and the other four *Dioscorea* species (*D. elephantipes*, GenBank accession: EF380353.1; *D. villosa*, GenBank accession: KY085893.1; *D. zingiberensis*, GenBank accession: KP899622.1; *D. rotundata*, GenBank accession: KJ490011.1) chloroplast genomes from GenBank were aligned using MAFFT v7 (Katoh & Standley, 2013) with default settings, assuming collinear genomes for the full alignment, and then we checked the small inversions through subsequent adjustment manually using Se-Al 2.0 (Rambaut, 1996). The nucleotide diversity of the chloroplast genome was conducted based on a sliding window analysis with the DnaSP v5.10 software (Librado & Rozas, 2009). The window length was set to 800 bp, with a 200 bp step size. Any large structural events, such as gene order rearrangements and IR expansions/contractions, among the nine species were ascertained.

### Phylogenetic reconstruction

Phylogenetic relationships were reconstructed using nine *Dioscorea* species chloroplast genomes and *Tacca chantrieri* was used as an outgroup. The entire chloroplast genome, LSC, SSC, and IR regions were used to construct phylogenetic trees based on the differentiation of molecular evolutionary rates in chloroplast genome regions.

The program ModelFinder was used to find the optimal substitution mode (Kalyaanamoorthy et al., 2017). We performed Maximum Likelihood (ML) analyses using RAxML v.8.1.24. The general time reversible + G model was chosen in all analyses with 1,000 rapid bootstrap replicates.

Bayesian inference (BI) of the phylogenies was implemented with MrBayes v.3.2.2 (Ronquist et al., 2012). A Markov Chain Monte Carlo Analysis was run for 10,000,000 generations with trees sampled every 1,000 generations, with the first 25% discarded as burn-in. The remaining trees were used to construct a 50% majority-rule consensus tree.

## Results

### Genome sequencing and assembly

Five *Dioscorea* species were sequenced to produce 53,889,722-81,562,406 raw reads (150 bp for average read length). The complete chloroplast genomes of *Dioscorea* are 428,514–3,050,140 with 838× to 5,944× coverage (Table 1). The accuracy of inverted repeat junction regions in assembled sequences were further confirmed by PCR amplification and Sanger sequencing with specific primers. The five *Dioscorea* cp genome sequences were then submitted to GenBank (accession numbers MG267378, MG267381–MG267384).

**Table 1:** Sampling and assembly information for the five *Dioscorea* species.

Species	ID	Raw data no.	Mapped read no.	Precent of chloroplast genome reads (%)	Chloroplast gemome coverage (X)	Accession number
*D. aspersa*	LJW01	69,648,118	428,514	0.62%	838	MG267381
*D. alata*	LSC09	77,185,326	2,467,928	3.20%	4,834	MG267382
*D. bulbifera*	LAW08	53,889,722	1,140,614	2.12%	2,235	MG267383
*D. futschauensis*	MHW01	81,562,406	3,050,140	3.74%	5,944	MG267384
*D. polystachya*	MHW08	62,610,816	1,119,774	1.79%	2,192	MG267378

**Notes:**

W, wild.

C, cultivated.

### Genome size and features

The total chloroplast genome sizes of *Dioscorea* are 152,609 in *D. elephantipes* to 155,418 in *D. rotundata* (Table 2) with a pair of IR regions (25,464–25,576 bp) separated by an LSC region (82,777–85,600 bp), and an SSC region (18,806–19,038 bp). The overall GC content was 37–37.2%, indicating nearly identical levels among the *Dioscorea* chloroplast genome. The IR regions have a higher GC content (43.0%) than the LSC regions (34.9%) and the SSC regions (31.0%) (Table 2). The high GC content of the IR regions is possibly due to the high GC content of the four rRNA genes in these regions.

**Table 2:** Characteristics of the chloroplast genomes of nine *Dioscorea* species.

Genme features	*D. aspersa*	*D. alata*	*D. bulbifera*	*D. futschauensis*	*D. polystachya*	*D. elephantipes*	*D. villosa*	*D. zingiberensis*	*D. rotundata*
Size (bp)	153,337	153,161	153,075	153,946	153,243	152,609	153,919	153,970	155,418
LSC length (bp)	83,517	83,414	83,226	83,979	83,431	82,777	83,865	83,950	85,600
IR length (bp)	25,478	25,464	25,499	25,529	25,489	25,513	25,576	25,491	25,484
SSC length (bp)	18,864	18,819	18,851	18,909	18,834	18,806	18,902	19,038	18,850
Total genes	112	112	112	112	112	112	112	112	112
Protein coding genes	78	78	78	78	78	78	78	78	78
tRNA genes	30	30	30	30	30	30	30	30	30
rRNA genes	4	4	4	4	4	4	4	4	4
Overall GC content (%)	37.0	37.0	37.0	37.2	37	37.2	37.2	37.2	37.2
GC content in LSC (%)	34.8	34.8	34.8	35.0	34.8	34.9	35.0	35.1	35.2
GC content in SSC (%)	31.0	31.0	30.8	31.2	30.9	31.2	31.2	31.2	30.9
GC content in IR (%)	43.0	43.0	43.0	43.0	42.9	43.0	43.0	43.0	42.9

**Figure 1:**
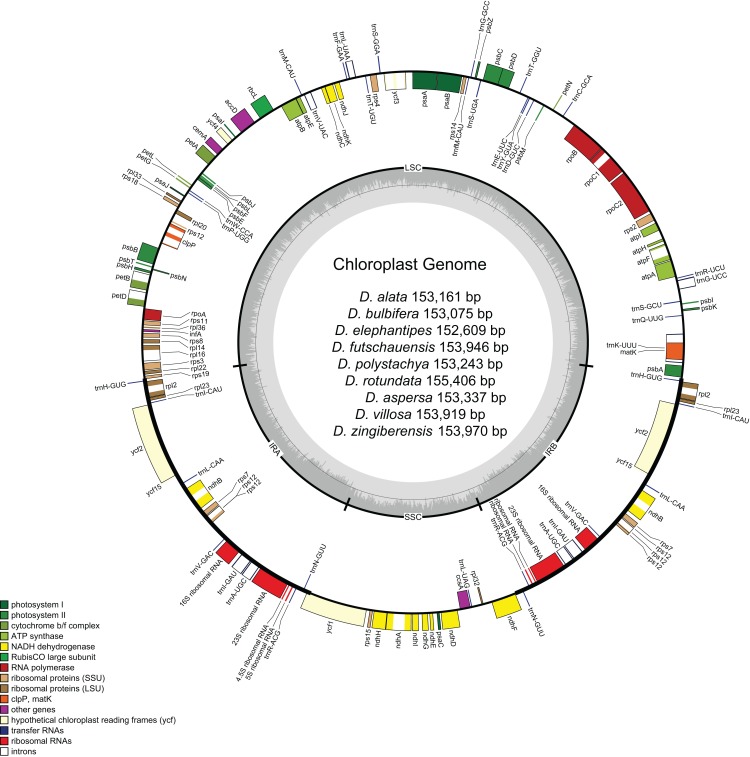
Gene maps of chloroplast genomes of Dioscorea. Genes on the inside of the large circle are transcribed clockwise and those on the outside are transcribed counter clockwise. The genes are color-coded based on their functions. The dashed area represents the GC composition of the chloroplast genome.

The *Dioscorea* chloroplast genomes encoded 112 unique genes, with 79 protein-coding genes, 29 tRNA genes, and 4 ribosomal RNA genes (Fig. 1; Table S1). The LSC region comprised 62 protein-coding and 22 tRNA genes, and the SSC region was composed of 12 protein-coding genes and one tRNA gene. Six protein-coding (*ndhB*, *rpl23*, *rps7*, *rps12*, *ycf2*, *rpl2*), eight tRNAs (*trnA-UGC, trnH-GUG, trnI-CAU, trnI-GAU, trnL-CAA, trnN-GUU, trnR-ACG, and trnV-GAC*) and four rRNA genes (*rrn4.5, rrn5, rrn16, rrn23*) were found be duplicated in IR_A_ and IR_B_. There are 17 intron-containing genes, of which 12 were protein coding genes and five were tRNA genes. *clpP* and *ycf3* had two introns, whereas the rest contained single introns. The *rps12* is a trans-splicing gene, having the first exon in the LSC region and the second and third exons in the IR regions.

### Repeat analysis and single sequence repeats

**Figure 2:**
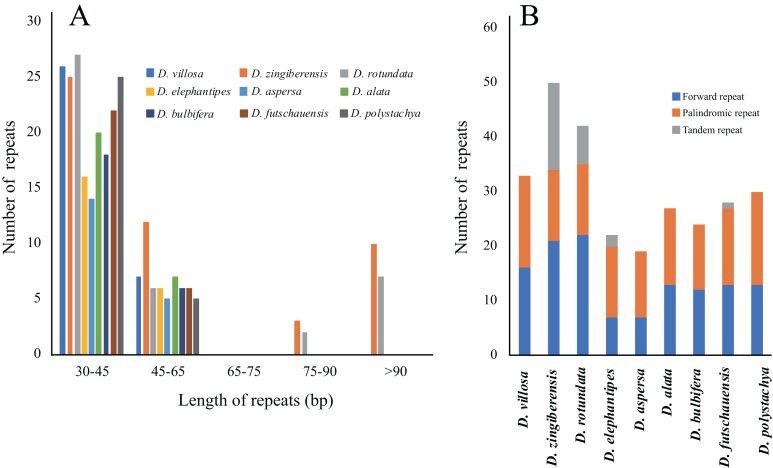
Analysis of repeated sequences in nine *Dioscorea* species. (A) Number of repeated sequences by length; (B) Number of types repeated three times in the nine chloroplast genomes.

We used REPUTER and Tandem Repeats Finder for the identification of the repeats, which are at least 30 bp. In total, 275 repeats were detected in the nine *Dioscorea* chloroplast genomes (Fig. 2). Each *Dioscorea* chloroplast genome contained 19–50 repeat sequences, including 7–72 forward repeats and 12–17 palindromic repeats. There were many fewer tandem repeats in *Dioscorea*. The tandem repeats analysis detected one in *D. futschauensis*, two in *D. elephantipes*, and 16 in *D. zingiberensis*. Among these repeats, most repeats (70.18%) were 30–45 bp in length, while those with more than 75 bp were few (8%).

**Figure 3:**
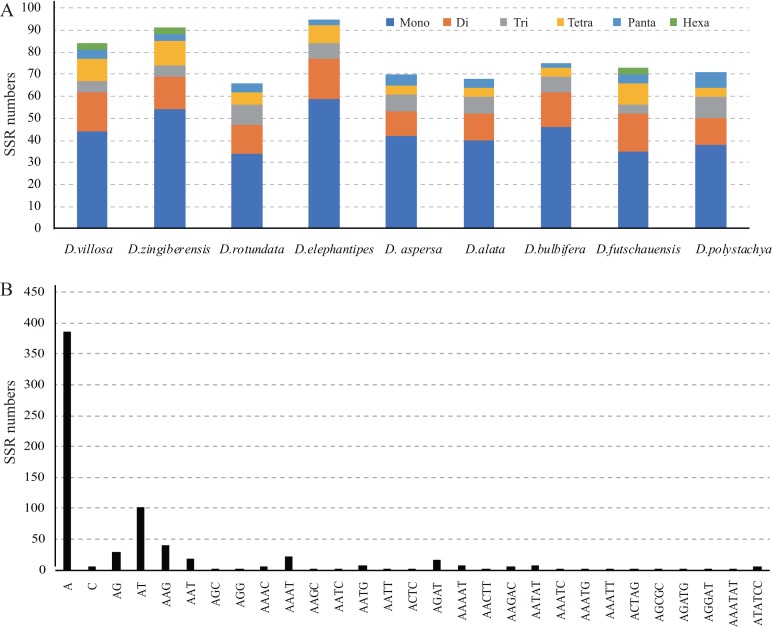
Analysis of simple sequence repeats (SSR) in the chloroplast genomes of nine *Dioscorea* species. (A) Number of different SSR types detected in the nine genomes; (B) Number of identified SSR motifs in different repeat class types.

Moreover, SSRs of the nine chloroplast genomes were analyzed (Fig. 3). Among them, *D. elephantipes* (95) had the most SSRs, and *D. rotundata* (66) had the least. The majority of the SSRs in these chloroplast genomes consist of mono- and dinucleotide repeat motifs, varying from 34 in *D. rotundata* to 59 in *D. elephantipes* for mononucleotide repeats, while dinucleotide repeats varied from 11 in *D. aspersa* to 18 in *D. villosa*, and *D. elephantipes* (Fig. 3A). Trinucleotide and tetranucleotide SSRs were the second most common, ranging from four in *D. futschauensis* to 10 in *D. polystachya* for trinucleotide repeats, while tetraucleotide repeats varied from 4 to 11. Furthermore, three hexanucleotide repeats were found in *D. villosa*, *D. zingiberensis*, and *D. futschauensi*. SSRs were particularly rich in AT in the *Dioscorea* chloroplast genomes. The majority of SSRs in all species were A/T mononucleotides (Fig. 3B).

### Phylogenetic analysis

**Figure 4:**
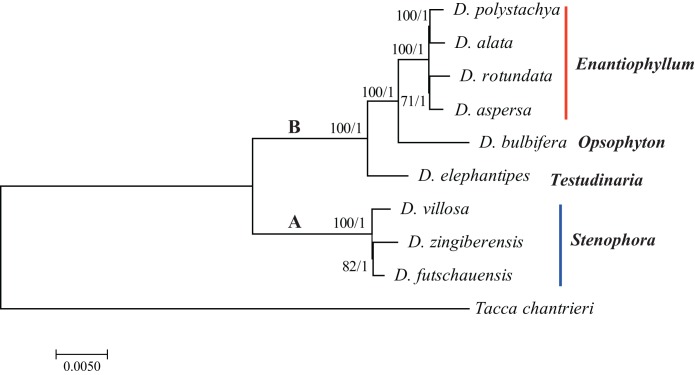
Phylogenetic tree reconstruction using maximum likelihood, and Bayesian inference methods based on the complete chloroplast genome sequences. ML topology shown with ML bootstrap support values/Bayesian posterior probability listed at each node.

Phylogenomic analysis within *Dioscorea* were reconstructed using ML and BI methods. The topologies based on analyses using the two methods were highly concordant in each dataset, as well as phylogenetic trees with moderate-to-high support (Fig. 4; Fig. S1). For the nine *Dioscorea* species, they were grouped into two branches: **A** clade (including *D. villosa, D. zingiberensis*, and *D. futschauensis*) and **B** clade (including *D. elephantipesn, D. bulbifera, D. aspersa, D. rotundata*, *D. alata*, and *D. polystachya*). In **B** clade, *D. elephantipes* was the sister of *D. bulbifera* and sect. *Enantiophyllum*. The short branch lengths in the sect. *Enantiophyllum* and *Stenophora* suggest rapid radiation evolutionary history in these clades. The phylogenetic positions of these groups are in agreement with recent studies (Hsu et al., 2013; Wilkin et al., 2005).

### Genome divergence and divergence hotspot

Nine complete *Dioscorea* chloroplast genomes were used for comparative analyses. The genome size of *D. rotundata* (155,418) is the largest of these, and this difference was mostly attributed to variation in the length of the LSC region (Table 2). *Dioscorea* had the highest chloroplast genome homologies, while there were more common linear relationships among the other plants (Jiang et al., 2017; Xu et al., 2017).

Sequence identity comparisons among the nine chloroplast genomes were plotted using the program mVISTA with the annotated *D. elephantipe*s sequence as a reference (Fig. S2). The whole aligned sequences showed high similarities with only a few regions below 90%, suggesting that *Dioscorea* chloroplast genomes were rather conserved. In addition, the IRs regions were more conserved than the single-copy regions, and noncoding regions exhibited a higher level of divergence than coding regions in the complete chloroplast genomes.

**Figure 5:**
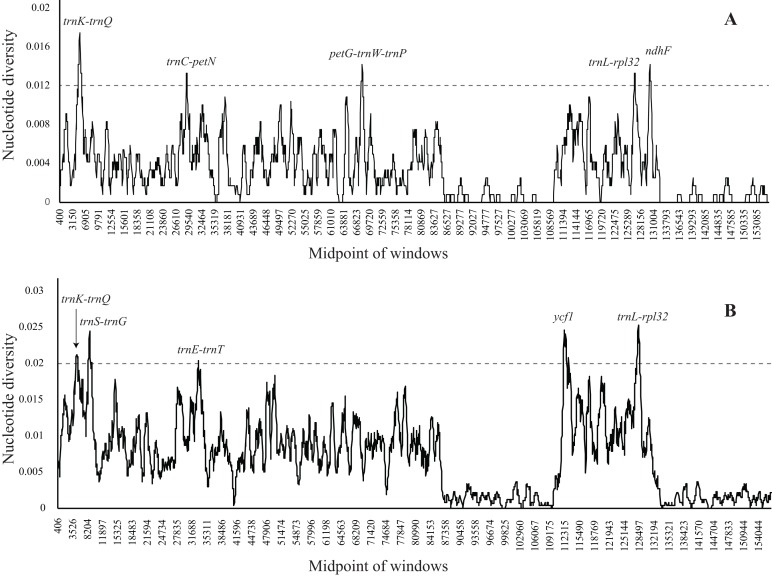
Sliding window analysis of the *Dioscorea* chloroplast genomes (window length: 800 bp; step size: 200 bp). (A) Nucleotide diversity of **A**-clade dataset; (B) Nucleotide diversity of **B**-clade dataset. *X*-axis: position of the midpoint of a window; *Y*-axis: nucleotide diversity of each window.

**Figure 6:**
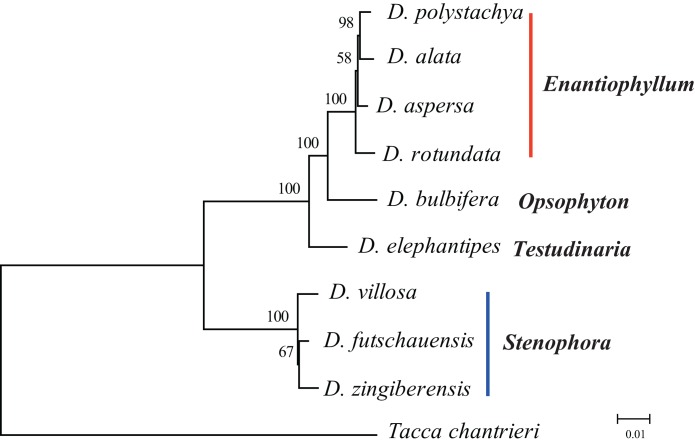
Phylogeny of the nine *Dioscorea* species constructed using eight regions of highly variable sequences. Numbers above nodes are support values with ML bootstrap values.

To identify the divergence hotspot regions, nucleotide diversity values within 800 bp in the nine *Dioscorea* chloroplast genomes were calculated with the DnaSP v5.10 software (Fig. 5). According to the phylogeny results, nucleotide diversity was calculated in both **A** clade and **B** clade. In the *Dioscorea* chloroplast genomes, nucleotide diversity values within 800 bp varied from 0 to 0.0175 in **A** clade and 0 to 0.02533 in **B** clade, respectively. The average value of nucleotide diversity was 0.00334 in **A** clade and 0.00926 in **B** clade. We identified eight divergence hotspot regions that could be utilized as potential makers to reconstruct the phylogeny and plant identification in this genus. Two are in the coding regions *ndhF* and *ycf1*, and six are in the intergenic regions (*trnK-trnQ*, *trnS-trnG*, *trnC-petN*, *trnE-trnT*, *petG-trnW-trnP*, and *trnL-rpl32*). Five of these regions lie in the LSC, and three are in the SSC. Phylogenetic tree reconstruction using ML methods based on eight divergence hotspot regions showed moderate-to-high support (Fig. 6), similar to the tree topology based on the complete chloroplast genomes. *trnK-trnQ*, ***trnS-trnG***, and *trnE-trnT* had higher resolution among the eight hotspots (Fig. S3).

## Discussion

### Chloroplast genome sequence variation and evolution

In this study, five new chloroplast genome sequences of *Dioscorea* were sequenced using Illumina sequencing technology, and another four additional *Dioscorea* species chloroplast genomes from GenBank were simultaneously taken into consideration for comparative analyses. Gene and intron content are highly conserved among land plant plastomes (Dong et al., 2013), although losses have been identified in several angiosperm lineages. The chloroplast genomes of *Dioscorea* species are structurally conserved and no rearrangement events were detected in this study. Meanwhile, the genome divergence was low. mVISTA results revealed high similarities among chloroplast genomes, suggesting that the *Dioscorea* cpDNAs were rather conserved. Similar results have been reported previously in angiosperms chloroplast genomes, and the lower sequence divergence in the IR regions compared to the SSC and LSC regions is possibly due to copy corrections between IR sequences by gene conversion (Zhang et al., 2017). Furthermore, the divergent regions included *trnK-trnQ*, *trnS-trnG*, *trnC-petN*, *trnE-trnT*, *petG-trnW-trnP*, which were consistent with previous reports that these divergent regions were mostly present in the SSC and LSC regions and showed a trend toward more rapid evolution (Rogalski et al., 2015; Scarcelli et al., 2011; Shaw et al., 2007).

We identified 275 repeats in the nine *Dioscorea* chloroplast genomes, which include dispersed, palindromic, and tandem repeats. Previous studies have shown that repeat sequences may play roles in rearranging sequences and producing variation through slipped-strand mispairing and illegitimate recombination (Morrison, 2009; Ochoterena, 2009). Furthermore, the presence of these repeats indicates that the region is a crucial hotspot for genome reconfiguration. The majority were distributed in non-coding regions, which were the highly variable regions in the chloroplast genomes (Asaf et al., 2017). Additionally, these repeats are an informative source for phylogenetic studies (Le Flèche et al., 2001; Rokas & Holland, 2000).

### Potential DNA barcodes for yam

Because of the more than 600 species, great morphological diversity, dioecism, and small flowers in *Dioscorea*, its DNA barcoding and taxonomy is still difficult to unravel after many years (Hsu et al., 2013; Wilkin et al., 2005). The chloroplast genome markers *matK*, *rbcL*, and *psbA-trnH* have been widely served as universal barcoding applications in plants (Borisenko, Sones & Hebert, 2009; Hollingsworth, Graham & Little, 2011); however, these markers had extremely low discriminatory power (Sun et al., 2012). *matK* only successfully identified 23.26%, compared with 9.30% for *rbcL* and 11.63% for *psbA-trnH* (Sun et al., 2012). Therefore, the development of reliable and effective DNA barcodes with high percentage of variable sites is very important for *Dioscorea*.

In the chloroplast genome, indels and SNPs were not random but clustered as “hotspots” (Scarcelli et al., 2011). Those “hotspots” regions were defined as highly variable locies. Based on the nine compared *Dioscorea* cpDNAs, eight highly variable regions (*trnK-trnQ*, *trnS-trnG*, *trnC-petN*, *trnE-trnT*, *petG-trnW-trnP*, *ndhF*, *trnL-rpl32*, and *ycf1*) are identified (Fig. 5). *ndhF*, *trnL-rpl32*, and *ycf1* have been the focus of DNA barcodes and hypervariable markers for phylogenetic reconstruction in previous studies (Dong et al., 2015; Shaw et al., 2007).

Recently, *ycf1*, a gene essential for plant viability and encodes Tic214, which was a vital component of the Arabidopsis TIC complex, was more focused on DNA barcoding and phylogeny (Dong et al., 2012, 2015; Xu et al., 2017). It was more variable than the *matK* and *rbcL* in most plant lineages (Dong et al., 2015; Neubig et al., 2009). *Ycf1* exhibited high variability in **B** clade of *Dioscorea* (Fig. 5). *NdhF* was widely used in tree of life and was considered as a variable coding gene in chloroplast genome (Chen et al., 2016; Kim & Jansen, 1995; Prather, Ferguson & Jansen, 2000). It exhibited relatively high variability in **A** clade of *Dioscorea* (Fig. 5)*. Rpl32-trnL* and *trnS-G* showed considerable length variation across taxa and a high level of positional variability (Shaw et al., 2007). The *trnC-petN* was part of *trnC-trnD* IGS which was divided into three IGS, *trnC-petN*, *petN-psbM*, and *psbM-trnD*. This region appeared to contain large indels, and the length of this IGS was variable (615–989 bp) across taxa. The nucleotide diversity of *trnK-trnQ* was 0.0175 in **A** clade of *Dioscorea* (Fig. 5), which were the highest markers. The *trnE-trnT* and *petG-trnW-trnP* were less used in plant phylogeny and DNA barcoding before. Therefore, further work on investigating whether these markers could recommend as effective, specific barcodes for *Dioscorea* species is necessary.

### Phylogenetic analysis

Recently, plastome information has provided a large amount of data for improving phylogenetic resolution. Chloroplast genome sequences have been widely used for the reconstruction of phylogenetic relationships among plant lineages (Burke et al., 2016; Dong et al., 2017; Du et al., 2017; Sun et al., 2016). Phylogenetic analyses of plant species using a small number of loci might frequently be insufficient to resolve evolutionary relationships, particularly at low taxonomic levels (Hilu & Alice, 2001; Majure et al., 2012). Many previous phylogenetic work based on whole chloroplast genomes have been used to resolve difficult phylogenetic relationships among closely related species (Carbonell-Caballero et al., 2015; Dong et al., 2017) and to enhance our understanding of the evolutionary relationships among angiosperms (Goremykin et al., 2013; Luo et al., 2016). Phylogenetic relationships of *Dioscorea* were estimated using several chloroplast DNA markers (*rbcL*, *matK*, *trnH-psbA*). However, they are insufficient to resolve evolutionary relationships (Gao et al., 2008; Hsu et al., 2013; Wilkin et al., 2005). Our phylogenetic analysis based on the dataset of complete chloroplast genomes indicated very clear internal relationships of *Dioscorea*. The phylogenetic trees indicated that the nine species of *Dioscorea* clustered into two groups. Furthermore, the phylogenetic trees indicated clear internal relationships of sect. *Enantiophyllum* and *Stenophora*, which may result from ancient, rapid radiations. However, our study was just a glimpse of phylogenetic relationships within the genus *Dioscorea*, and we will sequence more *Dioscorea* chloroplast genomes to estimate solid phylogenetic relationships and enhance our understanding of the evolution and diversification of characteristics of *Dioscorea* in the future.

## Conclusions

We assembled, annotated and analyzed five new complete chloroplast genome sequences of *Dioscorea*, and compared them with four chloroplast genomes from GenBank. The repeated sequences, microsatellites and eight highly variable regions (*trnK-trnQ*, *trnS-trnG*, *trnC-petN*, *trnE-trnT*, *petG-trnW-trnP*, *ndhF*, *trnL-rpl32*, and *ycf1*) were identified in *Dioscorea* chloroplast genome. Phylogenetic relationships of the *Dioscorea* species inferred from chloroplast genomes obtained high support even at the shortest internode. Furthermore, chloroplast genomic resources, in combination with other informative molecular markers from the mitochondrial and/or nuclear genomes, could be useful for phylogenetic analysis and species identification of the genus *Dioscorea*, as well as for population genetics.

## Supplemental Information

10.7717/peerj.6032/supp-1 Supplemental Information 1 Fig. S1. Phylogeny of *Dioscorea* species inferred from ML and BI analyses of different chloroplast genome sequences. ML topology shown with ML bootstrap support values/Bayesian posterior probability listed at each node.

10.7717/peerj.6032/supp-2 Supplemental Information 2 Fig. S2. Visualization alignment of chloroplast genome sequences of *Dioscorea*. VISTA based similarity graphical information portraying the sequence identity of *Dioscorea* with reference *D. elephantipe*s chloroplast genome. Grey arrows above the alignment indicate the orientation of genes. Purple bars represent exons, blue ones represent introns, and pink bars represent non-coding sequences (CNS). A cut-off of 50% identity was used for the plots. The Y-scale axis represents the percent identity within 50%–100%.

10.7717/peerj.6032/supp-3 Supplemental Information 3 Fig. S3. ML trees of *Dioscorea* species based on eight variable chloroplast regions, showing the resolutions of the loci in the group. The figures above the lines are the bootstrap values for the clades.

10.7717/peerj.6032/supp-4 Supplemental Information 4 Table S1. List of genes found in the Dioscorea chloroplast genomes.

10.7717/peerj.6032/supp-5 Supplemental Information 5 Sequences for the five Dioscorea species.

10.7717/peerj.6032/supp-6 Supplemental Information 6 Coed line.

## Additional Information and Declarations

### DNA Deposition

The following information was supplied regarding the deposition of DNA sequences: The five Dioscorea chloroplast genome sequences were deposited in GenBank accession numbers: MG267378, MG267381, MG267382, MG267383, MG267384.

### Data Availability

The following information was supplied regarding data availability: The raw data can be found at GenBank SRA: SRR7062185, SRR7062349, SRR7062357, SRR7062294, SRR7062296.
