# Supplementary figures and images for: Complete chloroplast genome sequences of Dioscorea: Characterization, genomic resources, and phylogenetic analyses
Source: PeerJ. 2018 Dec 4;6:e6032. doi: 10.7717/peerj.6032 (PMC6284424; doi:10.7717/peerj.6032)

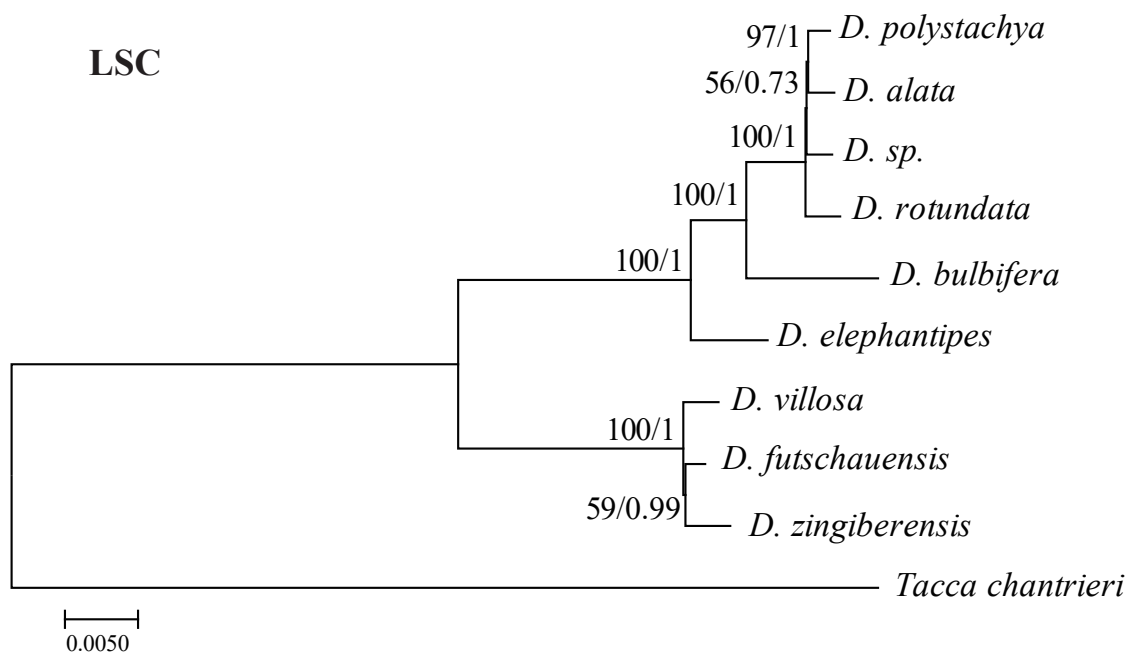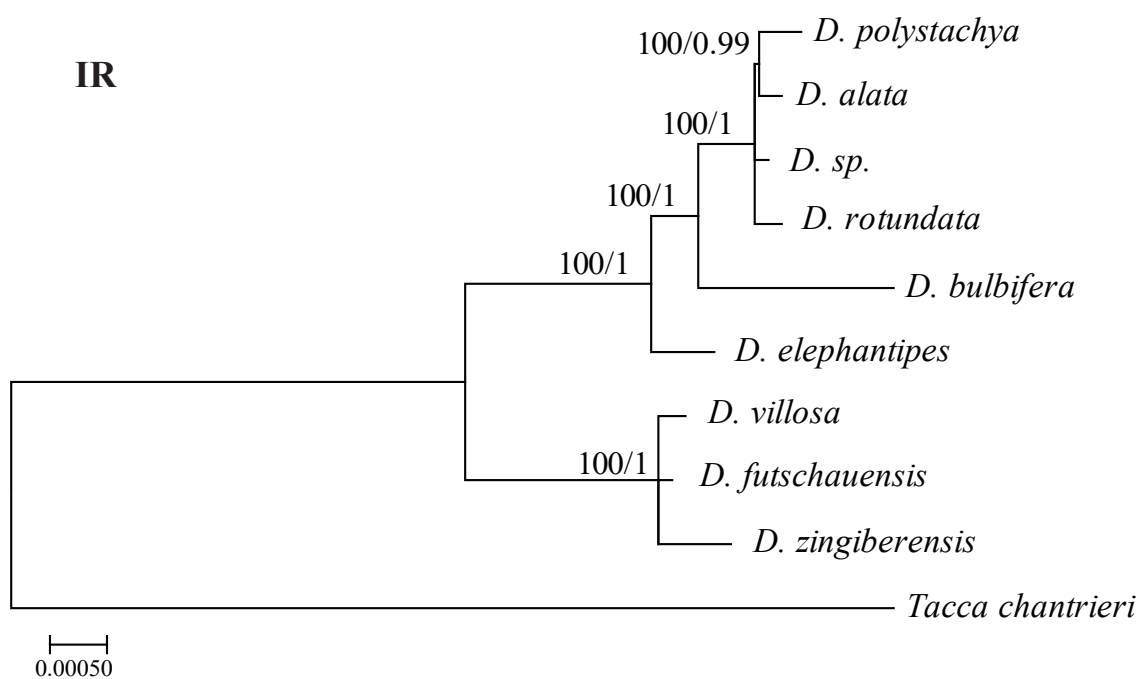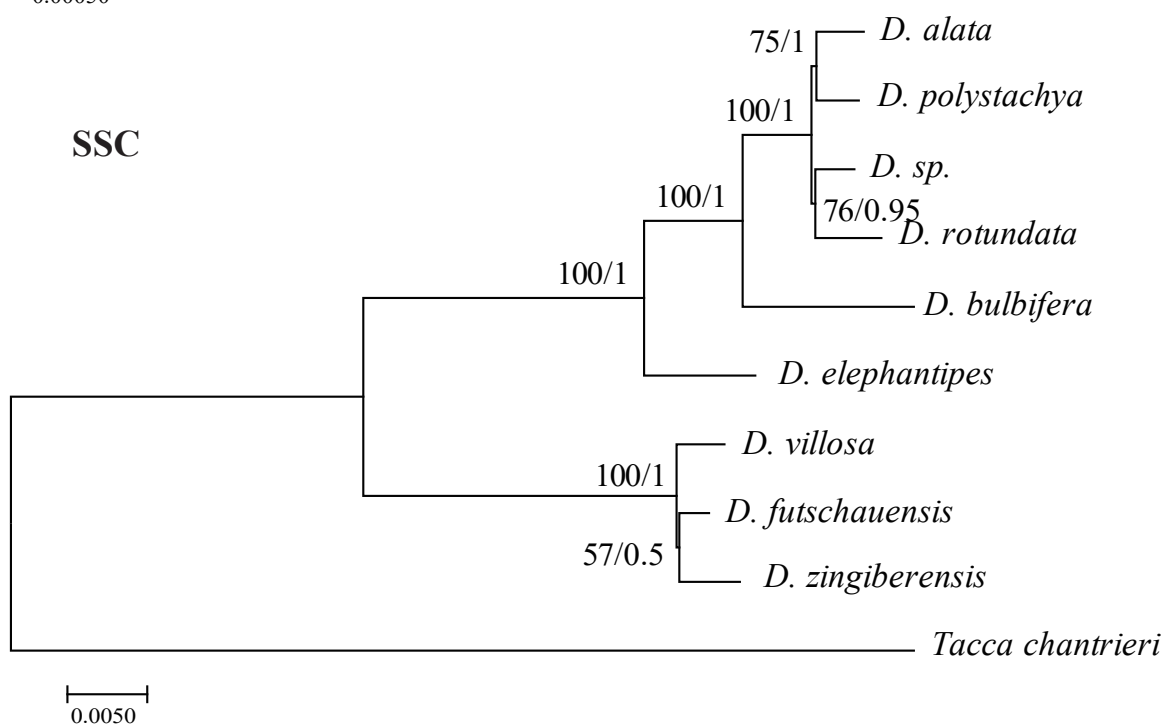

Supplement: Supplemental Information 1 — ML topology shown with ML bootstrap support values/Bayesian posterior probability listed at each node. [file peerj-06-6032-s001.pdf]

***trnK-trnQ***

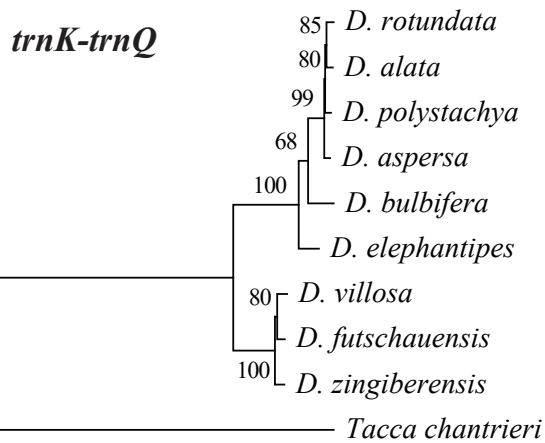

***trnC-petN***

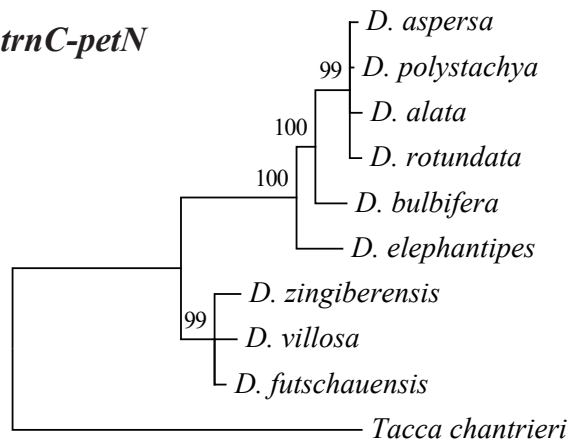

***petG-trnW-trnP***

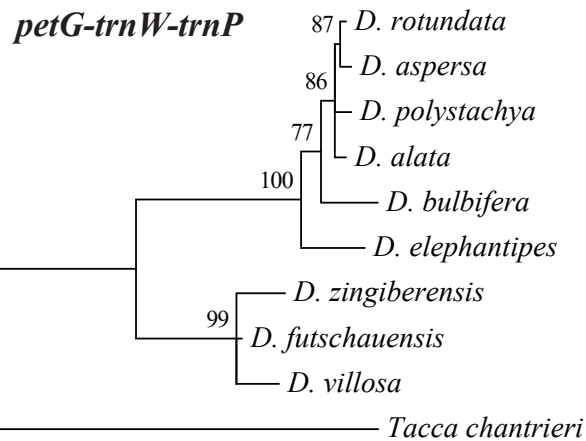

***trnL-rpl32***

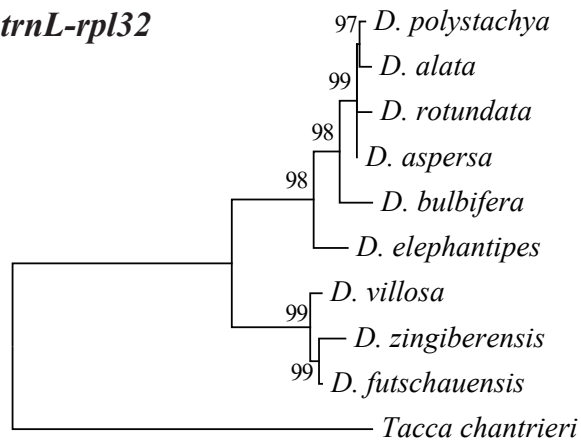

***ndhF***

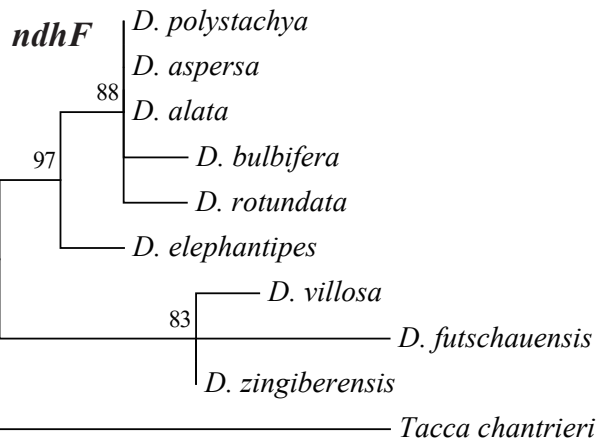

***trnS-trnG***

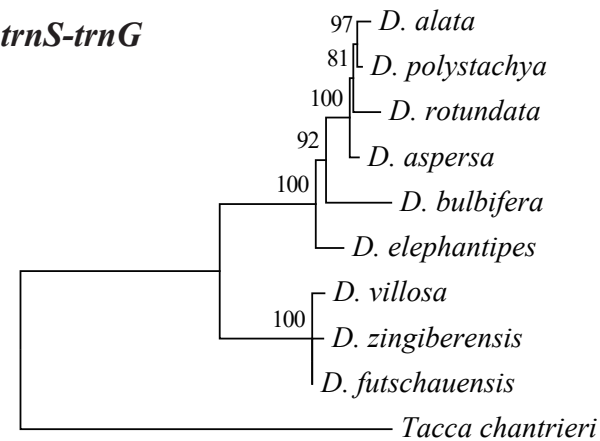

***trnE-trnT***

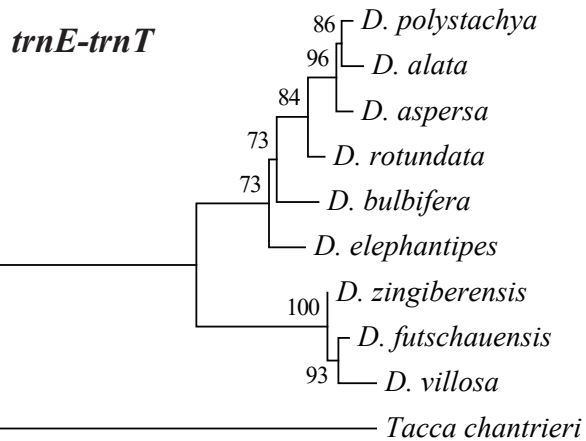

***ycf1***

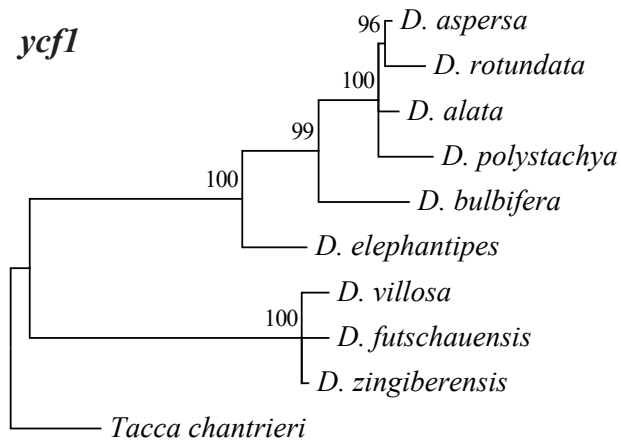

Supplement: Supplemental Information 3 — The figures above the lines are the bootstrap values for the clades. [file peerj-06-6032-s003.pdf]
