# Supplementary material for: Complete chloroplast genome sequences of Dioscorea: Characterization, genomic resources, and phylogenetic analyses
Source: PeerJ. 2018 Dec 4;6:e6032. doi: 10.7717/peerj.6032 (PMC6284424; doi:10.7717/peerj.6032)
Supplement: Supplemental Information 2 — VISTA based similarity graphical information portraying the sequence identity of Dioscorea with reference D. elephantipes chloroplast genome. Grey arrows above the alignment indicate the orientation of genes. Purple bars represent exons, blue ones represent introns, and pink bars represent non-coding sequences (CNS). A cut-off of 50% identity was used for the plots. The Y-scale axis represents the percent identity within 50%–100%. [file peerj-06-6032-s002.pdf]

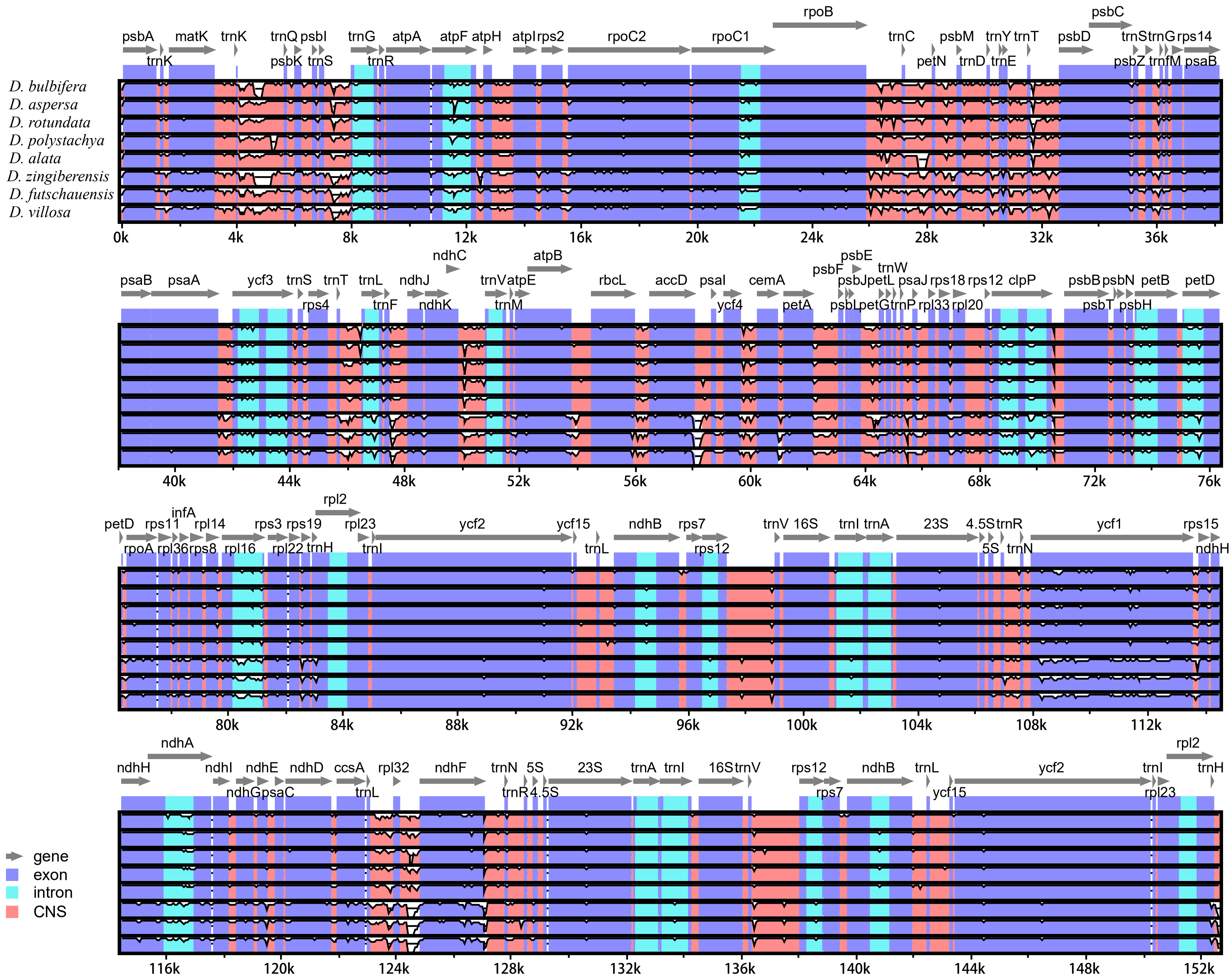
D. bulbifera
D. aspersa
D. rotundata
D. polystachya
D. alata
D. zingiberensis
D. futschauensis
D. villosa
psbA trnK matK trnK trnQ psbK psbI trnS trnG trnR atpA atpF atpH atpI rps2 rpoC2 rpoC1 rpoB trnC petN psbM trnD trnY trnE trnT psbD psbC trnS psbZ trnG trnfM rps14 psaB
0k 4k 8k 12k 16k 20k 24k 28k 32k 36k
psaB psaA ycf3 trnS rps4 trnT trnL trnF ndhJ ndhK ndhC trnV trnM atpE atpB rbcL accD psaI ycf4 cemA petA psbF psbE psbJ psbL petL petG trnW trnP psaJ rpl33 rps18 rpl20 rps12 clpP psbB psbT psbN psbH petB petD
40k 44k 48k 52k 56k 60k 64k 68k 72k 76k
petD rpoA rps11 rpl36 infA rps8 rpl14 rpl16 rps3 rpl22 rps19 rpl2 trnH rpl23 trnI ycf2 ycf15 trnL ndhB rps7 rps12 trnV 16S trnI trnA 23S 4.5S 5S trnR trnN ycf1 rps15 ndhH
80k 84k 88k 92k 96k 100k 104k 108k 112k
ndhH ndhA ndhI ndhG ndhE psaC ndhD ccsA trnL rpl32 ndhF trnN trnR 5S 4.5S 23S trnA trnI 16S trnV rps12 rps7 ndhB trnL ycf15 ycf2 trnI rpl23 rpl2 trnH
116k 120k 124k 128k 132k 136k 140k 144k 148k 152k
gene
exon
intron
CNS
