# Supplementary material for: Complete chloroplast genome sequences of Dioscorea: Characterization, genomic resources, and phylogenetic analyses
Source: PeerJ. 2018 Dec 4;6:e6032. doi: 10.7717/peerj.6032 (PMC6284424; doi:10.7717/peerj.6032)
Supplement: Supplemental Information 6 [file peerj-06-6032-s006.docx]

Code line:

1. NGS QC Tool Kit (code line: IlluQC_PRLL.pl -pe lib_pe1_left.fastq lib_pe1_right.fastq 2 5 -p 8 -l 70 -s 20)
2. SPAdes 3.6.1（code line: spades.py -k 95 --only-assembler --disable-gzip-output --pe1-1 lib_pe1_left.fastq.gz --pe1-2 lib_pe1_right.fastq.gz -o spades.output）
3. BLAST(-evalue le-10 –perc_identity 60 –qcov_hsp_perc 50)
4. MAFFT v7（code line: mafft -auto in.fasta > out.fasta）
5. RAxML v.8.1.24 (code line: raxmlHPC-PTHREADS -T 4 -f a -s dataset.phy -m GTRGAMMA -p 12345 -x 12345 -N1000 -n raxml.output)
